# Supplementary figures and images for: microbeSEG: A deep learning software tool with OMERO data management for efficient and accurate cell segmentation
Source: PLoS One. 2022 Nov 29;17(11):e0277601. doi: 10.1371/journal.pone.0277601 (PMC9707790; doi:10.1371/journal.pone.0277601)

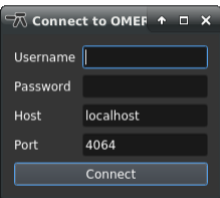

(a) OMER login

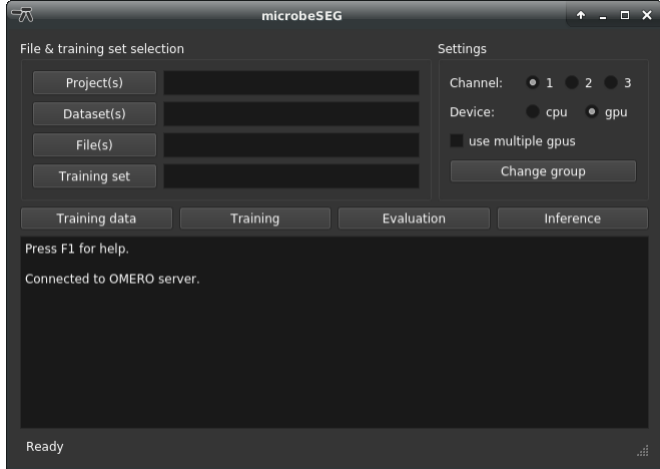

(b) Main menu

Supplement: S1 Fig — (PDF) [file pone.0277601.s001.pdf]

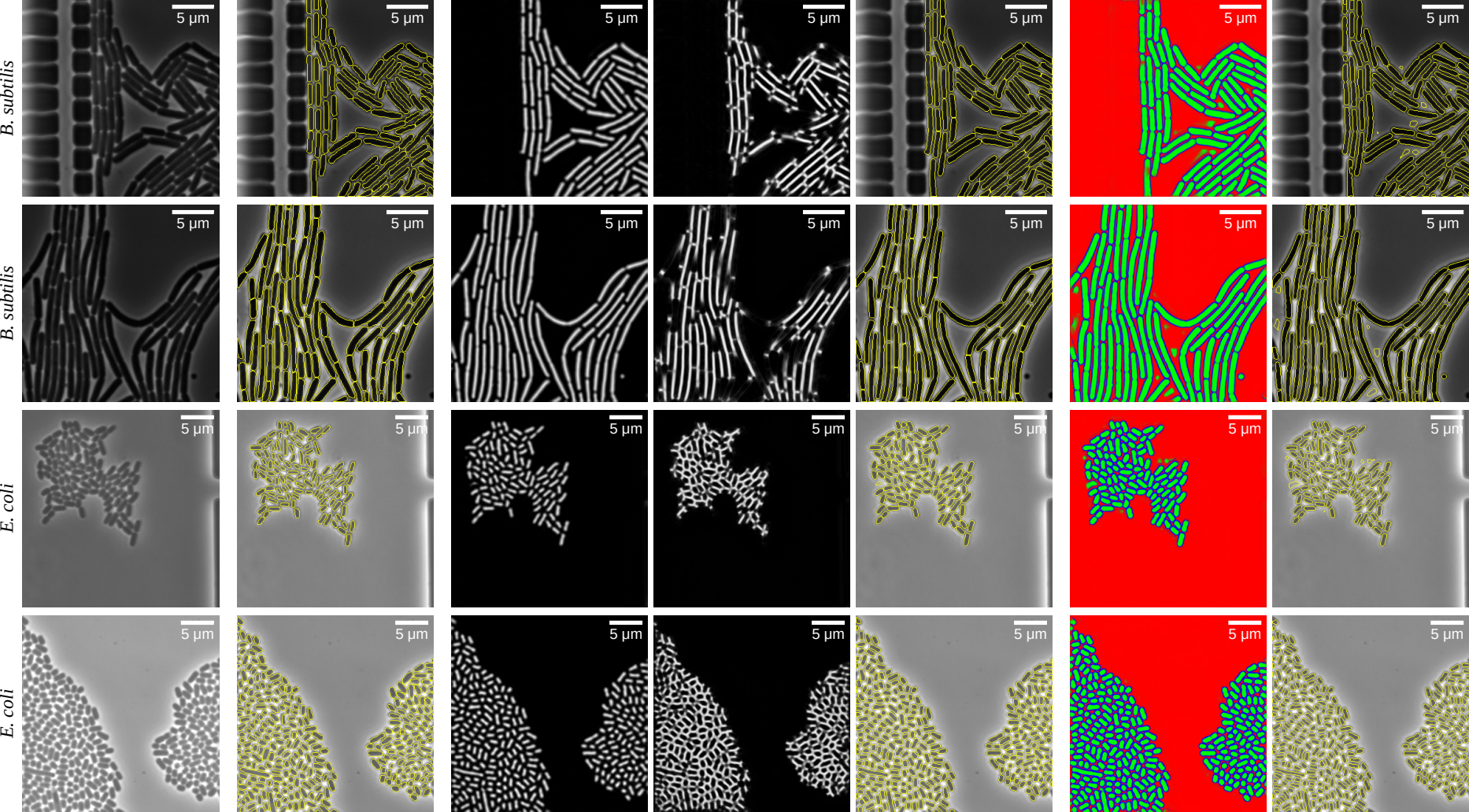

Supplement: S2 Fig — Shown are raw and post-processed predictions of the median distance method microbeSEG model (45 min) and of the boundary method microbeSEG model (45 min) reported in Table 1. Multi-channel predictions are color-coded (red: background, green: cell interior, blue: cell boundary). (PDF) [file pone.0277601.s002.pdf]
